# Evidence for Env-V2 sieve effect in breakthrough SIV _MAC251_ infections in rhesus monkeys vaccinated with Ad26/MVA and MVA/Ad26 constructs

**DOI:** 10.1186/1742-4690-9-S2-O32

**Published:** 2012-09-13

**Authors:** S Sina, S Tovanabutra, E Sanders-Buell, A Bates, M Bose, S Howell, G Ibitamuno, M Lazzaro, A O'Sullivan, J Lee, T Cervenka, J Kuroiwa, K Baldwin, DH Barouch, M Robb, R O'Connell, NL Michael, JH Kim, M Rolland

**Affiliations:** 1 BIDMC, Harvard Medical School, and Ragon Institute, Boston, MA, USA; 2 U.S. Military HIV Research Program/Henry M. Jackson Foundation, Bethesda, MD, USA

## Background

We had previously shown that rhesus monkeys receiving Ad26/MVA and MVA/Ad26 vaccines expressing SIV_SME543_ were protected against SIV_MAC251_ challenge (doi:10.1038/nature10766). Protection was associated with Env-specific binding ELISA antibody responses, including V2-specific antibodies.

## Methods

We amplified 66 sequences from the SIV _MAC251_ challenge stock, and 409 near-full length genomes from 13 vaccine and 13 control monkeys. A series of pre-specified phylogenetic and statistical tests for sieve effects was performed.

## Results

The mean pairwise AA diversity among the 66 SIV_MAC251_ Env sequences was 0.38%, and they differed from the vaccine strain SIV _SME543_ (Env) by 21.94%. The repeated low-dose challenge resulted in infections with an average of 1.7 founder variants - with no evidence that the vaccine restricted the number of variants (p = 0.813). We explored whether the vaccine induced a sieve effect, i.e. whether breakthrough viruses differed between the vaccine and control groups. There was no difference for full-length Env sequences. Focusing on Env segments preferentially recognized by vaccinated monkeys in antibody arrays, we identified a sieve effect in the Env-V2 segment AA163-193: sequences from vaccinated animals were more divergent from the vaccine SIV_SME543_ or from the challenge stock SIV_MAC251_ than sequences in control animals (p ≤ 0.002).

## Conclusion

The sieve effect in Env-V2, combined with Env-V2-specific binding antibodies identified as a correlate of protection against SIV _MAC251_ acquisition in the study, provide evidence supporting the importance of protective responses directed against the Env-V2 region.

